# From Community to Meta-Community Mental Health Care

**DOI:** 10.3390/ijerph15040806

**Published:** 2018-04-20

**Authors:** Nick Bouras, George Ikkos, Thomas Craig

**Affiliations:** 1 King’s College London, Institute of Psychiatry, Psychology and Neuroscience, De Crespigny Park, P.O. Box 27, London SE5 8AF, UK; thomas.craig@kcl.ac.uk; 2 Royal National Orthopaedic Hospital NHS Trust, Brockley Hill, Stanmore, Middlesex HA4 7LP, UK; ikkos@doctors.org.uk

**Keywords:** mental health, community, meta-community, psychiatry

## Abstract

Since the 1960s, we have witnessed the development and growth of community mental health care that continues to dominate mental health policy and practice. Several high-income countries have implemented community mental health care programmes but for many others, including mostly low- and middle-income countries, it remains an aspiration. Although community mental health care has been positive for many service users, it has also had severe shortcomings. Expectations that it would lead to fuller social integration have not been fulfilled and many service users remain secluded in sheltered or custodial environments with limited social contacts and no prospect of work. Others receive little or no service at all. In today’s complex landscape of increasingly specialised services for people with mental health problems, the number of possible interfaces between services is increasing. Together with existing uneven financing systems and a context of constant change, these interfaces are challenging us to develop effective care pathways adjusted to the needs of service users and their carers. This discussion paper reviews the developments in community mental health care over the recent years and puts forward the concept of “Meta-Community Mental Health Care”. “Meta-Community Mental Health Care” embraces pluralism in understanding and treating psychiatric disorders, acknowledges the complexities of community provision, and reflects the realities and needs of the current era of care.

## 1. Policy and the Political Economic Context

Community mental health care arguably marked the beginning of changes in mental health services with a strong ideological impetus [1]. The size of the population in long-stay public asylums was dramatically reduced, and Community Mental Health Teams (CMHTs) were introduced to replace traditional psychiatrist-led hospital outpatient visits and provide more rapid access to multidisciplinary teams better equipped to provide a broad range of psychosocial interventions. In short, the intention was to provide “a full range of effective mental health care to a defined population, dedicated to treating and helping people with mental health problems in proportion to their suffering or distress, in collaboration with other local agencies” [2].

In developed societies, at the social and political level, community mental health care emerged during the post-World War II Christian/Social Democratic era when “well ordered societies” were a reasonable aspiration [3]. However, in recent decades, mainstream politics in many countries have moved away from these communitarian visions of better and more equal societies towards a far greater emphasis on individualism in terms of personal responsibility for well-being and economic and social success. This societal change is paralleled by the increasing dominance of free-market economics and widening economic inequalities that have been shown to be causally associated with poorer mental health [4]. It has contributed to a failure to invest adequately in “social” care such as sufficient affordable housing, welfare, and social support services. Reflecting this dominant zeitgeist of “individualism”, mental health problems are increasingly conceptualised as belonging to the patient and, therefore, fixable by changing their brain (biological psychiatry) or their mind (psychological therapies), rather than tackling the interpersonal and wider social conditions that are responsible for causing or maintaining illness.

## 2. Community Mental Health Care: The Reality

From the earliest days, concerns and fears were expressed about the feasibility and sustainability of community care, mostly by psychiatrists but also some patients, carers, and other members of the community. Early critics included Leona Bachrach, who criticized community care in the US [5] as having failed to address the needs of the diverse population of patients, and by Kathleen Jones in the UK, who disapproved of the corrosive effect of community care ideologies on psychiatry in so far as they led to precipitate reductions in inpatient beds [6]. The new community mental health services were soon faced with two challenges. First, how to provide comprehensive care for people with persisting disability outside the asylum and, second, a growing appreciation of the enormous scale of mental health problems in the general population, among patients in primary care and in medical and surgical departments of acute hospitals.

### 2.1. Care for People with Persisting Severe Disability

Somewhere approaching 15% of people with a first-episode psychosis remain chronically symptomatic [7], and a considerable number of those with a diagnosis of psychosis manifest comorbidity with substance abuse or mild intellectual disability, Asperger’s syndrome, or physical health problems, or some combination of these. Though this group represents a relatively small proportion of people receiving mental health care, they absorb as much as 50% of the mental health and social care budgets [8]. Similar findings of “low-volume, high-cost” groups have been found for people with intellectual disabilities and challenging mental health problems and/or offending behaviour [9]. 

In the UK, the number of beds for long term residential care is now at the highest level it has been since 1972 [10]. In part, the increase in beds probably reflects population growth and increasing numbers of people reaching old age, including those suffering from dementia. However, this does not account fully for the growth in numbers. One of the fastest growing psychiatric subspecialties in the UK since the advent of community care has been forensic psychiatry and locked long-stay units, hardly what community care aspirations had anticipated [11]. Similar evidence for “trans-institutionalisation” and the growth of forensic residential care is reported across Europe. Significant numbers of these complex patients also end up in prison. For example, it has been noted in the US that while the proportion of the population with mental disorders in hospitals and correctional facilities is about the same as it was over 40 years ago, the balance has radically shifted from, previously, 75% in mental hospitals and 25% in penal institutions to, now, just 5% in mental hospitals and 95% in penal institutions [12]. In the US and Brazil, mentally ill people in prisons are more severely disturbed than those in psychiatric hospitals [13].

### 2.2. Demand Outstripping Supply

The strand of community care that established CMHTs and other outreach services has clearly been successful in bringing many more people into contact with mental health care. Therefore, it was not long before the new Community Mental Health Centres (CMHC)s were being overwhelmed by referrals and began struggling to provide care for the severely mentally ill, while also meeting demands from a large hitherto mostly unrecognised population [14]. New liaison services in general hospitals identified considerable unmet need [15] and there was mounting concern about other groups which are ill-served (or not served at all) by community mental health services: children and adolescents with behavioural and developmental disorders, those with intellectual disabilities, new groups of immigrants and refugees, and others. Evidence also quickly accumulated to show that the new CMHTs had very little impact on hospitalisation, with protracted incarceration having been replaced by a “revolving door” of repeated admissions followed by disengagement from treatment and subsequent relapse. 

In short, the high hopes that community care, linked with the best treatments, would result in significant improvements in long-term recovery and fewer relapses have not materialised. In the UK at least, successive legislation and service planning can be regarded as an attempt to deal with this explosion of conspicuous morbidity and the seemingly impossible task of reducing reliance on hospitalisation for severe mental illness. The one-stop shop model of the CMHT has fractured into a plethora of specialised teams that now encompass: services for people in acute and chronic states of distress, those experiencing a first episode of psychosis, people in crisis, and those who are deemed in need of treatment but who are reluctant to engage with care. Others include specialist services for people with developmental disorders but normal intelligence, eating disorders, autism spectrum disorders and adults with ADHD, liaison psychiatry, and so on.

Many of these developments have emerged on the back of both a sound rationale and even some evidence of efficacy, but with little regard as to how the different teams and services should relate to each other, resulting in a proliferation of complex difficult-to-navigate referral pathways. Though defended as a strategy to ensure that the “right” patients are seen by the right specialist service, in practice, fragmentation functions as a means of restricting patient flow across a clogged system.

A great deal of any lasting benefit of treatment is determined by the extent to which it helps the individual achieve a reasonable quality of life, and this typically depends on the availability and access to wider social provision in terms of adequate housing, work, and leisure opportunities. For all their faults, the best of the old asylums included occupational and leisure opportunities for the residents. Most regrettably, when they closed, the rate of employment for people with severe mental illness—whether voluntary or remunerated—declined [16]. The scant resources that had been provided for leisure activities were also removed, including the closure of many day care centres, mainly to reduce social care cost but typically presented as removing an institutional practice that was no longer necessary, as patients now resided in the community and, in theory, could access ordinary facilities in their new neighbourhoods.

The result has been social isolation and loneliness, as poignantly demonstrated in a survey of friendship among patients with schizophrenia in a coastal town in southern England [17]. Housing provision—typically, shared living that was thought appropriate in the 1980s—now seems woefully out of date and institutional in character. Even those people who survive independently often reside in impoverished, isolated, and socially excluding circumstances. What they need as a minimum is good social care (housing, income support, occupation, financial assistance), but these are the areas where budgets have been most deeply cut in the post-recession era. In the UK, even as the general National Health Service (NHS) healthcare budget remains relatively intact during the current global economic crisis, there has been a drastic reduction in funding of local authority-led provision, including public health care and social care service; among other consequences, this contributes greatly to delayed discharge from hospital [18,19].

## 3. Meta-Community Mental Health Care: Conceptual Foundations

In Greek, “meta” means “after”. Meta-community psychiatry and mental health care, thus, comes after and aims to address shortcomings of community care. The need for the concept arises partially from the multiplication of tightly boundaried services and interfaces described earlier, as well as the need to take constructive advantage of opportunities whenever they may arise [20]. It aims to be relevant not only to countries that are well advanced in community mental health care, but also to those that are in planning or implementing community care programmes. 

Conceptually, meta-community mental health care aims to strengthen the biopsychosocial model by underlining a pluralistic approach to understanding and treating mental illness. As argued by Kendler, “single broadly applicable explanatory laws are ill suited for our field”. He concludes that “biology will implement but not replace psychology within our explanatory systems. The iterative process of interactions between biology and psychology needed to achieve this implementation will deepen our understanding of both classes of processes” [21]. 

During the evolution of the human species there has been a dialectic process between the increasing size and abilities of the brain on the one hand, and the formation and increasing complexity of groups and socially achieved progress on the other [22,23]. It has not been a one-way influence from brain to socialisation. The human brain is biologically social. The ability to form larger and increasingly effective groups has been crucial to the increasing size and abilities of the brain. Uniquely, the human brain is specifically developed so that each one of us may be subject to interpersonal influences and predisposed to work together, even to display altruism for the advantage of the group. Any psychiatry that aspires to be scientific needs to put brain, mind, and social processes on an equal footing. We build on the work of neuro-philosophers [24] and evolutionary anthropologists [25], who have clarified the nature and centrality [26] of affect and confirmed the profound evolutionary significance of the social nature of human biology [27] to suggest that: “unlike neurologists, affect not the brain is the object of psychiatrists’ specialist medical expertise. Defined as feelings, emotions and agitations, affect integrates human responses and drives brain and body changes, thinking, perceiving, relating and acting. In no particular order, it depends on genes, evolution, culture, physiology, personal experience, social history, chance, meaning, the environment and a sense of self and others. Disturbance in any (combination) of these may lead to psychopathology ...” [28].

Attention to affect is consistent with the suggestion that, within a framework of social justice and evidence-based practice and robust professional ethics, “anything goes” in terms of conceptualising the “medical model”. Crucial to this approach is the recognition of the need to strengthen the evidence base about what works and in what context, but it needs to be combined with an equally strong values-based approach [29]. Importantly, what attention to affect adds is the need to focus on “what matters” to the patient—i.e., what fires them up or weighs them down, agitates or paralyses them—irrespective of whether this is due to biology, personal history, circumstances, or combination. The “personal recovery” clinical philosophy that calls for a greater focus on the goals and strengths, empowering rather than constraining the sufferer, is highly consistent with this formulation [30]. 

To pluralism, affect and “what matters” to the patient, as conceptual foundations of meta-community psychiatry, can be added “parity of esteem” between physical and mental health [31], i.e., ensuring that mentally ill people have as equitable access to effective treatment as do the physically ill, no matter where they may be placed. This requires confronting dogmatically or ideologically driven attempts which aim to limit services to certain settings. A good example of this is ensuring that necessary treatments are delivered to mentally ill people in prison, even if this means challenging existing prison regimes or invoking human rights legislation to ensure access [12]. A major and consistent focus on challenging discrimination is therefore essential.

Finally, sustainability must be added to complete the conceptual foundations of meta-community psychiatry. This refers to the need to ensure high-quality health care for future generations by balancing economic, environmental, and social demands to minimise disease burden and maintain health and to ensure that it is applicable across medicine [32]. 

## 4. Meta-Community Mental Health Care: Practical Foundations and Opportunities

Meta-community mental health care is consistent with the knowledge we already have of the principles of good mental health care, including the need for effective, accessible, efficient, and coordinated systems with meaningful service user participation and efforts to reduce the impact of stigma on access to care [33]. At its heart is the understanding of mental ill-health in its social context. Often, what people want is help with ordinary aspects of daily living and, in particular, access to the services and supports available to any citizen. As noted earlier, many of the aspirations of community care were founded on the recognition that mental illness can be caused, worsened, or maintained by damaging social processes in communities, neighbourhoods, schools, and families. These social causes can be prevented, or their effects mitigated, by interventions that address the social toxins (e.g., with milieu therapies, family work, clubhouses, and education and employment services) and by efforts to confront stigma and discrimination. 

There are several examples of developments that are illustrative of how the principles of meta-community mental health care are being realised:

- It is increasingly recognised that it is the duty of every mental health professional to strive to increase the understanding and acceptance by society of the reality of mental ill-health and the damaging consequences of prejudice, discrimination, and neglect. Although it is a dreadfully slow process, with much further to go, some evidence of progress is demonstrated through more sensitive media stories of mental health care and the public acknowledgement by some politicians and celebrities of their own mental health problems [34,35].
- A move away from reliance on residential shared accommodation with live-in staff, towards greater separation of housing provision from support to enable the individual to maintain a tenancy [36]. Although this approach goes back to the earliest days of community care, it is only in the last few years that a “housing first” approach has been widely applied to vulnerable groups, including homeless people with co-occurring complex mental illness and substance dependency [37,38]. This approach has not been shown to carry more risk of drop-out from wider care, or harm to self or others, and is preferred by patients over the more gradual “step down” models that are still the dominant provision for residential care of people with severe mental illness.
- Supportive employment services (also known as individual placement and support) that help people suffering from mental disorders to retain or return to work without lengthy pre-employment training and rehabilitation [39,40]. Despite considerable research evidence that these interventions are valued by patients, are cost effective, and benefit society, they are still poorly implemented in standard care, largely because they are seen as a social rather than health care provision and so are not adequately supported by health care funders.
- Personalised budgets that give the service user direct control over the purchasing of social and health care is yet another example. The patient and their care team(s) have a modest budget to supplement routine care and work together to identify an area of need but, given the additional resource, leave it (more or less) up to the patient to choose how best to meet this need. So far, such initiatives have mainly involved relatively small pilot programmes that have been open to the criticism that they are largely “tokenistic” or so hedged around by rules of access as to be of marginal relevance. Nevertheless, such schemes reflect a growing consensus that citizens should be put at the heart of public services and that this principle should also apply to mental health and social care [41].
- Socially focused interventions that may, in the long term, reduce the incidence of common mental disorders. Examples showing promise include parenting and schools’ programmes addressing conduct disorder in childhood (one of the strongest risk factors for adult disorders [42,43]. Other well-evidenced interventions include befriending support for older adults [44] and peer support in depression [45].
- Expansion and refinement of services that reach out to offender populations to divert those with a mental illness from police custody, courts, and prisons to appropriate mental health care [46,47].

Expanding interventions beyond the individually focused biological and psychological to reach into the social arena and to extend beyond traditional health care settings (i.e., extending to workplaces, schools, prisons, and asylum and refugee settings) will be challenging and will require more than the redeployment of existing staff. Therefore, manpower planning and staff training are an integral part of service development and sustainability. Targeted services offering specialist interventions may be better able to attract staff with specific skills and implement what we know are effective treatments. For example, “early intervention” teams for psychosis appear to have better outcomes in terms of relapse and functional outcomes [48,49] very possibly because they have been more successful at delivering evidence-based treatments, including combinations of pharmacological and psychological modalities. The time is also ripe for exploring alternatives to existing professional models of care, including looking at what can be delivered by less-skilled (and thus less expensive) staff. This is hugely important for sustainable mental health care. Interestingly, the lead for this has come from countries where trained professionals are rare, and much has been achieved through training lay members of the local community and generic health care staff to recognise and manage common mental disorder [50].

Of course, an evidence base for sustainable interventions for common mental disorders has also emerged from the developed world, where many of the interventions were first implemented. One relatively recent example in England and Wales is the Improving Access to Psychological Therapy (IAPT) programme. This draws upon psychology graduates who see IAPT as valuable experience for access to further specialist clinical psychology training. Within a brief period, these services had treated 1 million people, with reported recovery rates between 45% and 65%, and 45,000 people had moved off sickness benefit back to work [51]. While initially focused on depression and anxiety, the services are expanding to tackle personality disorders and psychoses, and to work with the elderly and with children and adolescents. Another evidence-based service development in the UK, also requiring evolving skills mix, is the Rapid Assessment Interface and Discharge service (RAID) approach [15] to liaison psychiatry. Both IAPT and RAID have led to services’ establishment and development after health economic assessments demonstrated their longer-term sustainability as well as clinical utility.

Less well evidenced than IAPT or RAID, but also of interest, are initiatives based on lay health workers, some of whom are in paid roles [52], and on peer support workers who bring the benefits of fellowship and shared experience and are able to model recovery and thus convey credible hope [53].

## 5. Conclusions

This article briefly reviews the implementation and delivery of community mental health care over recent years and argues that it has substantially failed to meet initial intentions, aspirations, and objectives. We suggest that the concept of meta-community mental health care may be more reflective of the current state of mental health service provision and offer a better orientation in thinking about future service development and manpower training. We do not offer a comprehensive account but aim to encourage reflection and debate. For example, we are fully aware that a lot needs to be worked out for older people in relation to meta-community mental health care. Meta-community health care principles and practice are evolving.

Fundamental principles include a pluralistic approach to mental illness, which is informed by reliable evidence but recognises affect, values-based practice and recovery and the important role of social care as central to the understanding and treatment of mental health problems; also, parity of esteem and equity of access to physical and mental health and patient-centred planning and sustainability. The training curriculum, professional values, and daily practice of psychiatrists should explicitly reflect such principles. Recent guidance by the European Psychiatric Association and the World Psychiatric Association is consistent with this [54,55]. Indeed, these documents may be conceived as beginning to map out the implications of what we have argued above for psychiatrists in terms of their professional practice during the emerging/prevailing meta-community era. Clinicians should be trained and be prepared to engage at all levels of practice and discourse, including social policy and public engagement. 

Mental health services should be provided where needed, including as traditionally conceived settings in the community and primary care, but also in electronic “space”, at school and place of work (increasingly important), general hospital and nursing homes, prison (increasing need in many countries), asylum centres, homeless hostels, and even in war zones. Meta-community mental health care is relevant to all settings where patients may be found, and the training of clinicians should also highlight the importance of liaison and smooth transition of care across different professionals, age groups, and settings [56,57]. Effective accessible and coordinated systems that run efficiently across sub-specialisations is in practice achievable now, given compatible electronic health records available across services.

The concept of meta-community mental health care can illuminate thinking and advance policy across countries with advanced community mental health programmes, as well as those embarking on reforms of established mental health services and those that may be starting from scratch, and act as a catalyst for a more realistic approach to mental health care. For example, it seeks to build on what has been successful about community care, whilst highlighting failures that require more attention. It implies that whilst attention to community-based services is necessary, it fails to address the care of the many mentally ill in prisons or general hospitals. It recognizes that the assumptions of community care are severely tested in our era of mass migrations, increasing authoritarianism and asylum seeking, and in the face of casualties (material, physical, and social) from proliferating wars. It also has the potential to address the difficulties that may arise as a result of the fact that, when the ideals of community psychiatry were first implicated, it was reasonable to assume that different categories of staff, their patients, and carers were from the same community, whereas now, in many developed countries at least, communication may be significantly impeded because they each live in “horizontal silos”, i.e., may have widely different ethnic, linguistic, and class heritages. In short, the concept of meta-community mental health care may help shake us out of complacency under the rubric of community psychiatry, emphasize the need for agility and adaptability, and offer renewed confidence that psychiatry and mental health services can improve across a wide range of practice and in diverse settings.
